# MetaTREE, a Novel Database Focused on Metabolic Trees, Predicts an Important Detoxification Mechanism: The Glutathione Conjugation

**DOI:** 10.3390/molecules26072098

**Published:** 2021-04-06

**Authors:** Angelica Mazzolari, Luca Sommaruga, Alessandro Pedretti, Giulio Vistoli

**Affiliations:** Dipartimento di Scienze Farmaceutiche, Università degli Studi di Milano, Via Mangiagalli 25, I-20133 Milano, Italy; luca.sommaruga8@gmail.com (L.S.); Alessandro.pedretti@unimi.it (A.P.); giulio.vistoli@unimi.it (G.V.)

**Keywords:** drug metabolism, glutathione conjugation, data accuracy, metabolic tree, MetaQSAR, classification algorithms, random forest

## Abstract

(1) Background: Data accuracy plays a key role in determining the model performances and the field of metabolism prediction suffers from the lack of truly reliable data. To enhance the accuracy of metabolic data, we recently proposed a manually curated database collected by a meta-analysis of the specialized literature (MetaQSAR). Here we aim to further increase data accuracy by focusing on publications reporting exhaustive metabolic trees. This selection should indeed reduce the number of false negative data. (2) Methods: A new metabolic database (MetaTREE) was thus collected and utilized to extract a dataset for metabolic data concerning glutathione conjugation (MT-dataset). After proper pre-processing, this dataset, along with the corresponding dataset extracted from MetaQSAR (MQ-dataset), was utilized to develop binary classification models using a random forest algorithm. (3) Results: The comparison of the models generated by the two collected datasets reveals the better performances reached by the MT-dataset (MCC raised from 0.63 to 0.67, sensitivity from 0.56 to 0.58). The analysis of the applicability domain also confirms that the model based on the MT-dataset shows a more robust predictive power with a larger applicability domain. (4) Conclusions: These results confirm that focusing on metabolic trees represents a convenient approach to increase data accuracy by reducing the false negative cases. The encouraging performances shown by the models developed by the MT-dataset invites to use of MetaTREE for predictive studies in the field of xenobiotic metabolism.

## 1. Introduction

The conjugation with glutathione (GSH) is a well-known reaction to detoxify electrophilic compounds [1]. Its relevance in toxicity mechanisms is owed to the fact that electrophilic molecules are responsible for drug-induced liver injury [2], which is a very frequent cause of the withdrawal of marketed drugs, as well as of the termination of clinical studies. Indeed, if not detoxified by GSH, electrophilic compounds can react with nucleophilic moieties within proteins and nucleic acids generating damaging covalent adducts that may cause several adverse effects such as eliciting immune responses [3].

The ability to predict in silico the metabolism of new chemical entities has attracted great interest in the last years since very common causes of drug failures (such as low efficacy, unsatisfactory pharmacokinetic profile, and toxicity) are often ascribable to an unfavorable impact on drug metabolism [4,5]. Most of the reported predictive studies focus on the redox reactions typically catalyzed by the CYP-450 enzymes [6], while only a few predictive tools for conjugation reactions were reported in the literature [7,8]. This lack of computational studies appears to be especially relevant for both glucuronidations [8,9] and, in particular, reactions with GSH [10] because these metabolic processes are very frequent in drug metabolism and, more importantly, play a key role in the detoxification processes [11].

The reactivity with glutathione is usually predicted by considering the presence of structural alerts, which allow potentially reactive molecules to be recognized [12]. Even though routinely applied, structural alerts can give incorrect predictions as they focus attention on the electrophilic moieties without evaluating the reactivity profile of the entire molecule [13]. Quantitative Structure-Activity Relationship (QSAR) analyses, mostly based on quantum mechanical descriptors, have been also proposed. However, they involve quite limited learning sets and have restricted applicability domains, so they are amenable only to predicting the reactivity of close congeners [14].

There are several reasons to explain the lack of general models to predict the reactivity to glutathione. First, the chemical variability of functional groups that can undergo conjugation with GSH is very broad and includes electrophilic moieties ranging from epoxides to α,β–unsaturated carbonyls, as well as thiols, disulfides, and peroxides [1]. Second, the reaction with GSH can be catalyzed by glutathione transferases (GST), but can also occur spontaneously, depending on the reactivity of the substrates and/or their capacity to fit the enzymatic pocket [15]. The last cause, common to all metabolic reactions, is the lack of truly accurate metabolic datasets. Most available databases are collected by automatic querying of online resources and, as such, they include a significant amount of inaccurate data and often combine xenobiotics with endogenous metabolic reactions [16].

Recently, we compiled and manually curated a metabolic database (MetaQSAR) by a meta-analysis of the specialized literature in the 2005–2015 years [17]. This project led to the generation of a database containing about 5000 metabolic reactions, whose reliability has been confirmed by several predictive studies [4,7,8,18]. Notwithstanding this, when used in classification predictive studies, even very accurate metabolic databases show a quite common problem concerning the definition of negative non-substrate molecules. Indeed, the positive class simply comprises all molecules for which the considered reaction (here the conjugation with GSH) is reported in the literature. In contrast, the definition of the negative class is more ambiguous, since it includes those molecules for which a given reaction is not described, but “the absence of evidence is not evidence of absence” [19]. Therefore, such a criterion for collecting the negative class is very questionable since the reaction can be unreported simply because the analyzed studies had different objectives and did not monitor the corresponding metabolites. This problem can lead to a significant number of false negatives that affect the reliability of the derived predictive studies.

Even though this problem cannot be completely resolved, we think that the number of false negatives can be significantly reduced if the data collection involves only publications reporting exhaustive metabolic trees. Indeed, one may suppose that studies comprising extensive metabolic trees are designed to detect the highest possible number of metabolites. Therefore, the unreported metabolites should be considered true negative non-substrates, apart from some minor metabolites that can be still undetected for technical limits of the analytical instruments [20].

On these grounds, we present MetaTREE, a new database on metabolic reactions including data extracted only from publications reporting exhaustive metabolic trees. Besides this, we report the first application of MetaTREE, by which we aimed to move forward towards the elucidation of the metabolic reactivity to glutathione as well as to investigate the benefits offered by this new kind of data curation procedure. The advantages of MetaTREE were assessed by working in parallel with two sets of data: (1) the MQ-dataset, extracted from MetaQSAR, and (2) the MT-dataset, extracted from MetaTREE. The accuracy of our predictions reaches encouraging standards in the field of metabolic models, with a Matthews correlation coefficient (MCC) of 0.67 and an area under the receiver operating characteristic curve of 0.94. Moreover, the best model performances are obtained thanks to the MT-dataset, proving that a higher level of data curation can improve the prediction accuracy. These results open further perspectives of application for MetaTREE in the field of metabolism prediction.

## 2. Results

### 2.1. Data Analysis

The data for this study were extracted from two databases internally developed: MetaQSAR and MetaTREE. The former is a database of metabolic reactions manually compiled from the specialized literature and critically annotated (described in detail in [17]). The latter is a new database of metabolic trees that results from a further data selection of the MetaQSAR database, to reduce the false negative rate within the database. From these databases, two binary classification datasets were extracted for the model generation, namely, MQ-dataset and MT-dataset (see Section 3.2). The MT-dataset is about half the size of the MQ-dataset and has the advantage to be well-balanced between the two classes.

**Figure 1:**
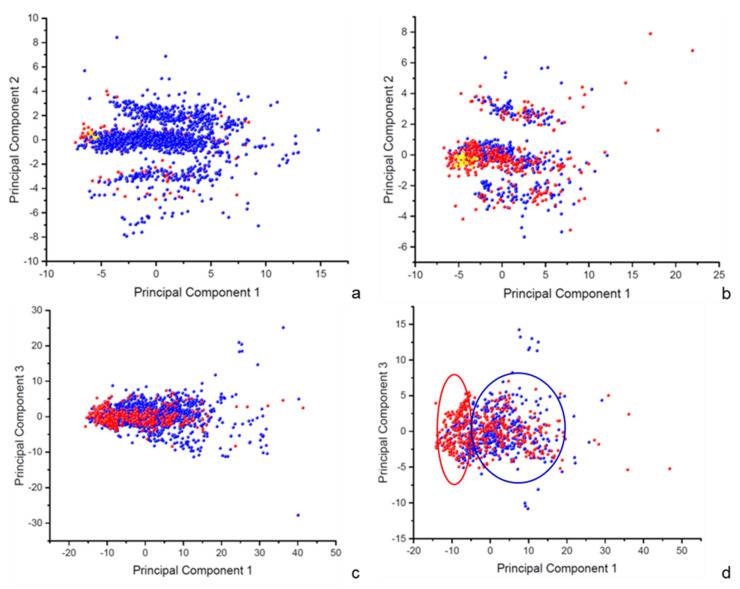
Scatter plots from PCA studies for MQ-dataset (**a**,**c**) for the first study and second study, respectively) and for the MT-dataset (**b**,**d**) for the first study and second study, respectively). “GSH substrates” and “GSH non-substrates” are displayed in red and blue, respectively. Yellow points correspond to the subset of known non-enzymatic GSH substrates.

Both datasets underwent principal component analyses (PCA) to characterize the chemical space covered by the training sets. These studies aim to extract new features that can reveal the presence of patterns inside the learning data and verify whether these patterns can have a predictive role for the reactivity toward glutathione. The percentage of variance expressed by the first three computed principal components are reported in Table 1 and the scores of the resulting principal components are depicted on scatter plots in Figure 1. Similar observations can be drawn for both the datasets, proving that they are, for the most part, a subset of each other and explore the same chemical space.

The first PCA involves 20 selected 3D-physicochemical and stereo-electronic properties and the first three generated principal components express a cumulative percentage of variance equal to 86.09% for the MQ-dataset and 86.77% for the MT-dataset. The first principal component results from the combination of relevant structural features, such as mass, volume, and surface, variously measured. As the consequence, molecules are spread out in the 2D-scatter plots according to their size, with smaller molecules at lower values of PC1 and larger molecules at higher values (Figure 1a,b). The second principal component mostly includes the electronic properties, consisting of the ionization potential and the HOMO and LUMO energies. This uncorrelated variable accounts for the ionization state of molecules and we observe a stratification along this component with three main clusters: positively charged molecules for negative values of PC2, negatively charged molecules for positive values of PC2, and neutral molecules around the 0 value. Accordingly, the small set of non-enzymatic substrates within both the datasets (in yellow), which are neutral, small, and soft electrophiles, are placed in the central cluster, at a low value for PC1. Despite this clusterization, no evident pattern can be observed that corresponds to the binary classification of molecules in “GSH substrates” and “GSH non-substrates” (in red and blue, respectively), therefore this unsupervised analysis does not assume a predictive capability.

The second PCA involves 127 1D-2D-3D descriptors, and despite the high number of correlated original variables, the first three generated principal components express a cumulative percentage of variance equal to 69.02% for the MQ-dataset and 66.21% for the MT-dataset. For this study, the interpretation of the main principal components is complicated by the high number of original variables. In both cases, molecules form a single big group in the scatter plots with a few outliers (Figure 1c,d). Only for the MT-dataset (Figure 1d), a slight separation of the two classes is observable along with the first principal component. There is a significant proportion of “GSH substrates,” in red, with a value of PC1 between −15 and −5, while “GSH non-substrates” are mostly placed at values of PC1 higher than −5. This finding can be interpreted as the first evidence that the criterion used to select molecules for MT-dataset is successful in catching the different chemical spaces covered by molecules able or not to react with glutathione. It is therefore expected that models based on MT-dataset will benefit from the additional step of data curation for accuracy and domain of applicability.

### 2.2. Model Building

The input matrices of the binary classification models include a large number of molecular descriptors (see under Methods for details) to provide to the models a wide variety of features among which to select the most informative ones. The pre-processing consists of two progressive steps that optimize the shape of the starting data and improve the performance of the models. The first step removes features based on the variance and excludes those for which none or a handful of observations differ from a constant value. This filter leads to a greater dimensionality reduction since it impacts mainly the 1024 ECFP descriptors. The second step refines the final size of the matrix by examining pairs of features and excluding the correlated ones.

Based on these refined data, models were generated by applying the random forest algorithm for binary classification. As detailed under Methods, a cross-validated grid search was conducted to optimize the algorithm hyperparameters. The internal validation was implemented on the preprocessed matrices by two methods. In the first, models were built on 70% of the dataset randomly selected and tested on the 30%, repeating this cycle 100 times and averaging results (MCCV). In the second method, the whole dataset was used for both training and testing, according to the LOO procedure. Since the MQ-dataset is slightly unbalanced, and this affects the predictive accuracy of the positive class for the corresponding models, a random undersampling procedure was also applied as a screening method to reduce the size of the negative class. In this procedure, 1270 molecules belonging to the non-substrate class were randomly selected and removed to obtain a starting dataset perfectly balanced between the two classes. A total of six models were then built, two for the MT-dataset and four for the MQ-dataset.

### 2.3. Model Evaluation

To evaluate the models from different perspectives, their performance was assessed by four metrics. The Matthews correlation coefficient (MCC) and the area under the receiver operating characteristic curve (AUC) were computed for an overall estimation, while precision and recall were utilized for a measure on the two classes separately. The MCC is a balanced metrics measuring the ability of the model to correctly classify all classes in the confusion matrix, while the AUC reveals the proportion between true positive and false positive at different threshold values. For the prediction of the single class, recall evaluates the number of instances that are correctly classified for each class, while precision accounts for the rate of correct predictions for each predicted class. Since the random forest models tend to favor the majority class of unbalanced datasets, the recall values of the minority class are often unsatisfactory, revealing a weakness of the model hidden by other metrics.

Table 2 shows the performances of the six generated models: four obtained by the MCCV and the LOO validation runs on both the datasets, two obtained by the MCCV, and the LOO validation runs on the MQ-dataset after random under sampling (US). The MCCV results are averaged over 100 evaluations and therefore are independent of the random split in training and test set before each evaluation. As a consequence of this, we can observe a high similarity between the MCCV performances and those obtained by the LOO models on the same dataset. Similarly, the US-MCCV model involves a procedure of data discarding that is repeated randomly before each of the 100 MCCV cycles so that the results are independent of the random deletion of learning data. On the contrary, the US-LOO performances depend on the set of negatives randomly selected to be discarded, leading to results that can be significantly different each time the model is run.

The best model, according to all the evaluation metrics, is the MCCV model built on the MT-dataset, with MCC equal to 0.67, AUC equal to 0.94, and sensitivity equal to 0.78. Even though, the reported models show limited differences in their overall metrics, the better performances of the MCCV model based on the MT-dataset can be better appreciated by focusing on the class specific metrics. Indeed, the MCCV model generated on the bigger and unbalanced MQ-dataset reaches very high precision and recall values for the NS class but, for what concerns the S class, the recall value does not improve the random prediction (specificity = 0.97, sensitivity = 0.55). Stated differently, the MCCV model based on the MT-dataset proves successful in recognizing the glutathione substrates while the corresponding model based on MQ-dataset affords unsatisfactory performances which reduce the overall metrics (MCC = 0.63, AUC = 0.91). The US-MCCV model on the MQ-dataset proves successful in increasing the sensitivity to 0.78 but, as the effect of the performance flattening to a similar value, the global predictive ability of the model does not even reproduce that of the corresponding total models (MCC (total) = 0.63, AUC (total) = 0.91, MCC (US) = 0.62, AUC (US) = 0.89). Moreover, the US LOO model shows even lower performances, being the worse among the generated models (MCC = 0.61, AUC = 0.85).

**Figure 2:**
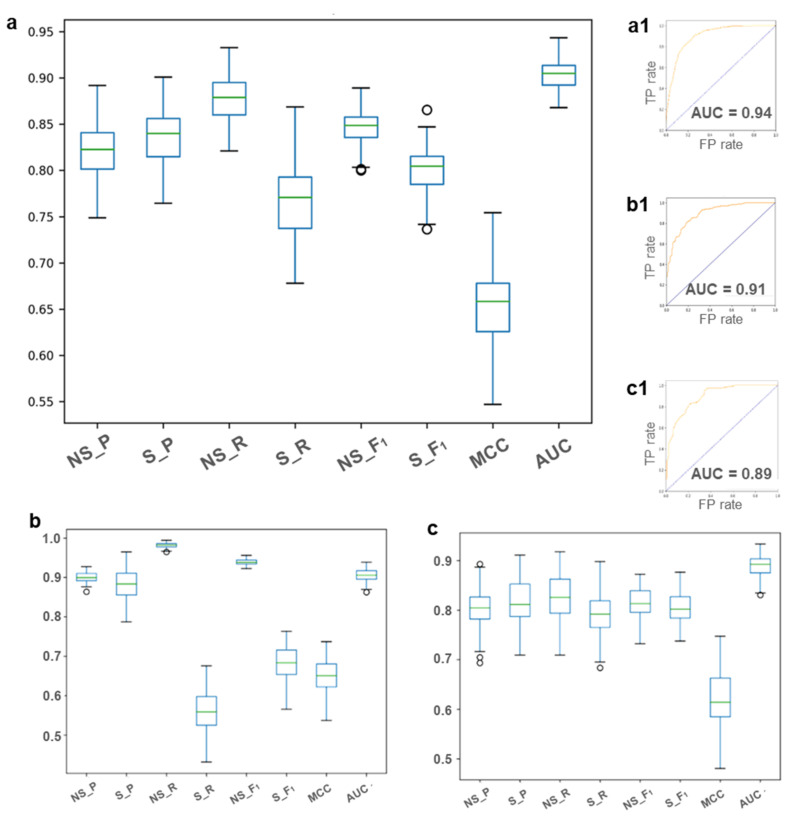
Box plots of the three MCCV models (**a**): MT-dataset, (**b**): MQ-dataset and (**c**): MQ-dataset after the random US, P: Precision, R: Recall, F_1_: F_1_ score, MCC: Matthew Correlation Coefficient) and the corresponding ROC curves (**a1**): MT-dataset, (**b1**): MQ-dataset and (**c1**): MQ-dataset after the random US, AUC: Area Under the Curve).

Figure 2 shows the box plots of the 3 MCCV models and the corresponding ROC curves. A considerable range of variability is observed within the 100 evaluations for nearly all of the performance measures. This is a sign of a wide structural variety inside the data, which confirms that our datasets explore a relevant proportion of the chemical space. Interestingly, this range is little only for the single class prediction of NS class for the MCCV model on MQ-dataset, as the consequence of the unbalanced dataset. Precision and recall metric values remain all near to 0.90 and 0.97, respectively, as the consequence of the higher precision offered by the random forest algorithm in respect to the majority class of an unbalanced dataset. The same behavior is indeed not retained when the random US procedure is applied (Figure 2c).

The last analysis involves the feature importance for the best performing models based on the MT-dataset. Table S1 (Supplementary Materials) lists the top 25 features for the LOO validated model and reveals the key relevance of the stereo-electronic descriptors. There are indeed four stereo-electronic parameters within the top 15 features. Their key role is further emphasized when considering that the input matrix included only 10 stereo-electronic descriptors. Notably, in all MT-dataset-based models generated both for hyperparameters’ optimization and by combining various sets of descriptors (results not shown), the core-core repulsion energy is always the most important feature. Overall, the stereo-electronic descriptors encode for the electrophilic nature of the collected molecules thus accounting for their propensity to reacting with the nucleophilic thiol function of GSH. Similar information can be encoded by the second feature WNSA-1 and related descriptors (WNSA-3, PNSA-1, PNSA-3, RNCS, and RPCS) which correspond to charge projections on the molecular surface [21]. Similarly, ATSc1 and ATSc3 represent autocorrelation descriptors based on atomic charges [22]. The top 25 features also include five physicochemical descriptors which mostly encode for the substrate lipophilicity and molecular size. They may describe the propensity of a given molecule to be metabolized as well as its capacity to fit the GST enzymatic cavities. Lastly, the top 25 features comprise five topological indices and three ECFP fingerprints which may encode for molecular shape and/or the presence of specific reactive moieties.

### 2.4. Applicability Domain Study

Models yield reliable predictions when their assumptions are valid and unreliable predictions when they are violated [23]. The Applicability Domain (AD) study defines the space where those assumptions are verified. One of the possible approaches for AD estimation is based on similarity analyses for the training set. Test compounds have a reliable prediction if they are similar enough to those used by the algorithm in the learning phase [24]. The similarity can be calculated according to many criteria. The performance of the model is plotted against the whole range of similarity in the dataset.

**Figure 3:**
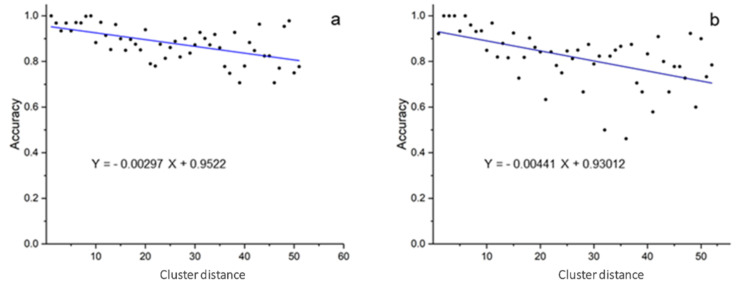
Graphical evaluation of the applicability domain by the plots of the accuracy (rate of correctly predicted instances) against the cluster distances for MT-dataset (**a**) and MQ-dataset (**b**).

As detailed under Methods, the AD was here assessed based on the predictions given by the LOO validation and the similarities computed by using the ESshape3D descriptors and by computing the value of the first nearest neighbor distance (NND). Based on the NND values the compounds were clustered, and for each cluster, the model predictive performance in terms of accuracy (rate of correctly predicted instances) on both datasets was plotted against the cluster distances (Figure 3).

A clear trend is shown in both the plots with predictive performances decreasing when the NND increases. This is consistent with what was expected: the predictive power of the model is directly correlated with the similarity between the test molecule and those in the training set. Some differences between the AD studies for the two utilized datasets are also observable. In detail, the accuracy values retrieved by the MT-dataset (Figure 3a), in comparison with those retrieved by the MQ-dataset (Figure 3b), are less scattered around the linear fit line and decrease less steeply with the decreasing of similarity (slope of 0.00441 against the slope of 0.00297). Both these behaviors are ascribable to a higher quality of the model generated from MT-dataset, which maintains the predictive accuracy more constant and linear and displays a larger applicability domain.

## 3. Materials and Methods

### 3.1. MetaTREE 

MetaTREE is a database of metabolic trees that is currently being drawn up in our research group. MetaTREE includes molecules and reactions reported in exhaustive metabolic studies that explore all the possible metabolites formed in the human body starting from a given substrate. The criterion to consider a metabolic tree as “exhaustive” and to be included in MetaTREE is that it should include at least second-generation metabolites. Hence, for the compilation of MetaTREE, we re-analyzed the same bibliographic sources of MetaQSAR [17] and discarded specific studies which did not meet the defined criterion since they are focused on a single metabolic reaction or a restricted group of biotransformations, as well as they investigate specific enzymes. In detail, we extracted 257 metabolic trees from 1461 papers, and we collected more than 1200 molecules with more than 1000 annotated metabolic reactions.

This database has the same structure as MetaQSAR and its annotations undergo the same classification system, which subdivides the metabolic reactions into 101 subclasses. The goal under the compilation of this new database is to easily provide datasets with a reduced false negative rate in the training data, with an improvement of performances for the resulting predictive models. A difference between the two databases is that MetaQSAR currently includes metabolites (and metabolic reactions) mostly belonging to the first and second generations, while MetaTREE, exploring exhaustive metabolic trees, also comprises metabolic data of higher generations. This difference can explain why the MT-dataset includes a higher number of GSH substrates compared to the MQ-dataset (see Section 3.2).

### 3.2. Dataset Collection

For the model generation, we searched MetaQSAR and MetaTREE databases and we collected two binary datasets, MQ-dataset and MT-dataset, respectively. The collected data were curated by excluding molecules with less than 8 atoms or bigger than 1000 Da, molecules with counter-ions, and molecules with rare elements. For what concerns the dataset extracted from MetaTREE, the second step of manual and expert curation was carried out to further reduce the rate of false negatives. Molecules, which are involved in high generation metabolic reactions and are reported as GSH non-substrates, while comprising electrophilic functional groups that can react with GSH, were discarded. This step is meant to exclude from the negative class those molecules which can structurally be plausible substrates for the GSH conjugation, but the corresponding conjugate might be undetected by the experiments for technical limits of the analytical instruments.

In the extracted datasets, molecules are classified as “GSH Substrates” (S) and “GSH Non-Substrates” (NS). According to the metabolic reaction classification of both databases, the former are molecules giving metabolic reactions coded as 24.01 or 24.02, the latter are molecules giving metabolic reactions other than coded as 24.01/.02/.03/04.

The first dataset (MQ-dataset), collected by searching MetaQSAR, includes 2106 molecules, of which 418 GSH substrates and 1688 GSH non-substrates, while the second one (MT dataset), extracted from MetaTREE, includes 980 molecules, of which 444 are GSH substrates and 536 are GSH non-substrates. Given the different criteria by which MetaTREE and MetaQSAR are collected, a key difference between the two resulting datasets is that MQ-dataset mostly includes metabolites of the first and second generations, while MT-dataset also comprises metabolic data of higher generation. Moreover, the curation step aimed to reduce the false negative rate in the training data mostly influences the class of the non-substrates. Indeed, the two datasets share only 325 non-substrates and the MT-dataset includes 211 non-substrates that are not included in the MQ-dataset and which mostly belong to high generation metabolic reactions. In contrast, the two datasets share 425 common GSH substrates with only 19 substrates comprised only in the MT-dataset. Table 3 shows an overview of the dataset composition.

### 3.3. Descriptors

Before descriptor calculation, the 3D structure of the collected molecules was optimized by applying a protocol of standardization, as follows: (i) the protonation state was simulated as compatible with the physiological condition (pH = 7.4) and tautomers were standardized by using the specific tool in the Maestro package, version 9.8.016 (Schrödinger Release 2016-4: Maestro, Schrödinger, LLC, New York, NY, 2016, USA); (ii) the conformational behavior was explored by a clustered procedure based on the Monte Carlo algorithms as implemented in the VEGA ZZ software [25], which generates 1000 conformers by randomly rotating the rotors and selects the lowest energy conformer; (iii) the 3D structures were refined by the semi-empirical method MOPAC 2016, [26] using the Hamiltonian operator PM7 [27] and preserving the existing chirality.

Various sets of molecular descriptors were investigated in this study and Table 4 provides a summary of those involved in our models.

The core set of descriptors comprised 20 descriptors, of which 10 are molecular physicochemical properties calculated by VEGA ZZ, and 10 are stereo-electronic molecular properties calculated by the semi-empirical simulations. In addition, a set of 127 more detailed 1D-, 2D-, and 3D-descriptors were investigated, including topological, constitutional, and geometrical descriptors, which were calculated by the software BlueDesc (http://www.ra.cs.uni-tuebingen.de/software/bluedesc/welcome_e.html, Eberhard Karls University of Tübingen, Germany). The missing values in the original matrix were replaced by the mean of each feature. The full list of these descriptors is reported in Supplementary Materials Tables S2 and S3.

Furthermore, Extended-Connectivity Fingerprints [28] (ECFP) were generated by the software GenerateMD version 15.3.2.0 (and the application for molecular descriptors were supplied by ChemAxon (http://www.chemaxon.com, Budapest, Hungary). These descriptors were extracted from the SDF files, setting the bond depth to 5 and computing the 1024-bit string form.

### 3.4. Principal Component Analysis

A Principal Component Analysis (PCA) was performed by the software Origin Pro (ORIGIN PRO version 2017, OriginLab Corporation, Northampton, MA, USA) and computing the eigenvalue decomposition of the data correlation matrix. Results were visualized by plotting the first three principal components (see Figure 1).

Two sets of descriptors were separately investigated (see Descriptors). The first study involved a set of 20 selected descriptors including 3D-physicochemical and stereo-electronic properties only; the second study focused on a set of 127 1D-, 2D-, and 3D-descriptors calculated by BlueDesc software. The missing values in the original matrix were replaced by the mean of each feature.

### 3.5. Pre-Processing and Parameter Optimization

To reduce model complexity, before each model was built, the input matrix was pre-processed to select the most informative descriptors. In the first step, the “near zero variance” descriptors were removed by exploiting the *VarianceThreshold* function from the Scikit-learn sub-library called *feature_selection* (http://scikit-learn.org/0.21/documentation.html). The best performing thresholds of minimum variance resulting from preliminary tests were equal to 0.0475 for the ECFP descriptors, which corresponds to a binary feature 95% composed of the same value and equal to 0.8 for all the other features. In the second step, all the non-binary descriptors undergo a normalization process by applying the *preprocessing.scale* function of Scikit-learn. Each feature value was standardized to have a mean value of 0 and a standard deviation of 1. Finally, in the third step, the remaining descriptors were checked for the presence of highly correlated features by computing the Pearson correlation coefficient between each pair of features and setting a threshold of 0.95.

To optimize three of the most important random forest hyperparameters (*max_features*, *n_estimators*, and *class_weight*) and to enhance the quality of predictions, a nested k-fold cross-validation was implemented to be run after the preprocessing on each input matrix, with k equal to 5 and the predictive performance measured by the F1 score metrics. The grid used for this hyperparameter optimization is shown in Table S4 (Supplementary Materials).

### 3.6. Model Building

Predictive models were built on the MQ-dataset (MQ-models) and the MT-dataset (MT-models). All models were generated by using the random forest [29] implementation from the machine learning package scikit-learn for Python 3.0 (http://www.python.org) running the code by JetBrains PyCharm Community Edition version 2016.2.3 (https://www.jetbrains.com/pycharm/ Prague, Czech Republic). In more detail, the specific function *RandomForestClassifier* imported from the Scikit-learn sub-library called ensemble was used. The hyperparameters were kept at their default values except for three parameters, i.e., *max_features*, *n_estimators*, and *class_weight*, the values of which were optimized by a five-fold cross-validated grid search (see above).

Model performances were validated by two internal validation methods: Monte Carlo Cross Validation (MCCV) and Leave One Out (LOO) validation. The MCCV consisted of a set of 100 repeated cycles of training and testing, each time randomly splitting the dataset in 70% and 30%, respectively. Given our matrices of n sample, the LOO validation method trained the model on n-1 samples and predicted the label of the 1 sample previously excluded. The prediction reliability was evaluated based on five measures: precision, recall and F1 Score for the individual class, as well as Matthews Correlation Coefficient (MCC) and AUC (area under the ROC curve) for the overall performance.

### 3.7. Applicability Domain Study

Applicability domain (AD) analysis based on LOO validation results was carried out to compare MQ-models and MT-models. The AD estimation was based on similarity analysis measured on ESshape3D descriptors, as implemented by the MOE software (Molecular Operating Environment; Chemical Computing Group Inc., 1010 Sherbooke St. West, Suite #910, Montreal, QC, Canada, H3A 2R7). This choice is justified considering that the ESshape3D fingerprints precisely account for the 3D structure of molecules and thus they can distinguish even between different stereoisomers. The ESshaped3D fingerprints comprise 122 integer value string descriptors that account for the 3D structure of molecules and can distinguish among different stereoisomers of the same compound.

The similarity between a given compound and the whole set of training compounds was evaluated by the value of the nearest neighbor distance (NND). The similarity matrix of the Euclidian distances (positive integer numbers) between each pair of compounds was computed and compounds were grouped in clusters according to their NND values.

## 4. Conclusions

This study continues our ongoing effort to enhance the accuracy of the metabolic data and improve the performances of the resulting predictive models. In the last decade, we embarked upon the compilation of a manually curated database (MetaQSAR), collected by a meta-analysis of the specialized literature in the years 2005–2015. This analysis was performed under the invaluable supervision of Prof. Bernard Testa, who critically reviewed all the screened publications. Such meticulous work led to a superior accuracy of the collected data as emphasized by the predictive studies based on MetaQSAR. Such an ongoing project has two possible extensions. On one hand, we are involved in a constant and critical updating of the databases by manually adding recently published papers in the metabolic field. On the other hand, we aim at further increasing its overall accuracy by revising and filtering the collected data, as here proposed.

Here, we try to further enhance the data accuracy by tackling the problem of false negative cases. Indeed, the selection of negative instances is an issue that very often affects the overall reliability of the collected learning sets. The negative instances are frequently based on absent data without probability parameters which can explain if the event can occur, but it is not yet reported, or it cannot occur. Drug metabolism is a typical field that experiences such a challenging situation. Indeed, predictive studies based on published metabolic data should consider that all metabolic reactions which are unreported are negative instances, but this is an obvious and coarse approximation because a lot of metabolic reactions can occur while being not yet published for a variety of reasons, starting from the simple motivation that they are not yet searched at all.

Hence, we propose to reduce the number of false negative data by focusing attention on the papers which report exhaustive metabolic trees. Such a criterion is easily understandable since this kind of metabolic study has the objective to characterize as many metabolites as possible. The so-developed new metabolic database (MetaTREE) showed a better data accuracy, as demonstrated by the enhanced predictive performances of the models obtained by using the MT-dataset compared to those of MQ-dataset. Indeed, the better performance reached by the MT-dataset for what concerns the sensitivity measure is due to a decrease in the false negative rate retrieved by the models. This result can be ascribed to the better selection of negative examples in the learning dataset, which should include a low number of molecules wrongly classified as “non substrates.”

Finally, the study emphasizes how accurate learning sets allow the development of satisfactory predictive models even for challenging metabolic reactions such as the conjugation with glutathione. Notably, the generated models are not based on the concept of structural alters but include various 1D/2D/3D molecular descriptors. They can account for the overall property profile of a given substrate, thus allowing a more detailed description of the factors governing the reactivity to glutathione. Even though the proposed models cannot be used to predict the site of metabolism or the generated metabolites, we can figure out two relevant applications. First, they can be used to rapidly screen large molecular databases to discard potentially reactive compounds in the early phases of drug discovery projects. Second, they can be used as a preliminary filter to identify the molecules that deserve further investigations to better characterize their reactivity with glutathione.

## Figures and Tables

**Table 1:** Results from the two PCA studies as applied to the MQ-dataset and the MT-dataset.

Study	No. Original Descriptors	Dataset	Principal Component	Variance (%)	Cumulative Variance (%)
First study	20	MQ-dataset	PC1	64.80	64.80
			PC2	14.04	78.84
			PC3	7.25	86.09
		MT-dataset	PC1	64.86	64.86
			PC2	14.57	79.43
			PC3	7.34	86.77
Second study	127	MQ-dataset	PC1	54.45	54.45
			PC2	7.92	62.37
			PC3	6.65	69.02
		MT-dataset	PC1	49.98	49.98
			PC2	10.56	60.54
			PC3	5.67	66.21

**Table 2:** Performances of the six developed predictive models for the two considered datasets. Both the entire MT- and MQ-datasets were used to obtain models by the MCCV, and the LOO validation runs. Due to its unbalanced nature, the MQ-dataset was also utilized to generate models by the MCCV and the LOO validation runs after random undersampling (US). For MCCV models and for MCC and AUC metrics, standard deviations are also reported.

Metrics	MT-Dataset MCCV		MT-Dataset LOO		MQ-Dataset MCCV		MQ-Dataset LOO		MQ-Dataset MCCV Random-US		MQ-Dataset LOO Random-US	
	NS ^a^	S	NS	S	NS	S	NS	S	NS	S	NS	S
Precision	0.83	0.84	0.81	0.84	0.90	0.87	0.89	0.88	0.81	0.82	0.76	0.78
Recall	0.88	0.78	0.88	0.78	0.97	0.56	0.97	0.56	0.83	0.78	0.78	0.75
MCC	0.67 ± 0.04		0.66		0.63 ± 0.04		0.63		0.62 ± 0.07		0.61	
AUC	0.94 ± 0.02		0.94		0.91 ± 0.02		0.89		0.89 ± 0.02			

(a) the molecules are classified as “GSH substrates” (S) and “GSH non-substrates” (NS).

**Table 3:** Datasets composition.

Class	MQ-Dataset	MT-Dataset	Shared Molecules
S	1688	536	325
NS	418	444	425
Total	2106	980	750

**Table 4:** Sets of descriptors used in the study with their dimensionality, the number of computed descriptors, the type of data, and the utilized software. The ESshaped3D fingerprints were used only for the applicability domain analyses.

Descriptor	Dimensionality Level	Number of Descriptors	Type of Data	Software
Physicochemical	3D	10	continuous	VEGA ZZ
Semi-empirical electronic	3D	10	continuous	MOPAC
Geometrical, topological, constitutional	1D/2D/3D	127	continuous	Bluedesc
ECFP	2D-topological	1024	boolean	ChemAxon
ESshaped3D	3D	122	fingerprints	MOE

## Data Availability

The data presented in this study are openly available in Zenodo repository doi: 10.5281/zenodo.4637110.

## Supplementary Materials

The following are available online, Table S1: List of the top 25 features for the LOO validated model based on the MT-dataset, Tables S2 and S3: Full lists of the involved descriptors, Table S4: Grid used for this hyperparameters optimization.
