# Supplementary material for: MetaTREE, a Novel Database Focused on Metabolic Trees, Predicts an Important Detoxification Mechanism: The Glutathione Conjugation
Source: Molecules. 2021 Apr 6;26(7):2098. doi: 10.3390/molecules26072098 (PMC8038802; doi:10.3390/molecules26072098)
Supplement: Supplementary file 1 [file molecules-26-02098-s001.pdf]

Supporting information for

MetaTREE, a novel database focused on metabolic trees, predicts an important detoxification mechanism: the glutathione conjugation

Angelica Mazzolari [1]*, Luca Sommaruga[1], Alessandro Pedretti [1] and Giulio Vistoli [1],

[1] Dipartimento di Scienze Farmaceutiche, Università degli Studi di Milano, Via Mangiagalli, 25, I-20133 Milano, Italy
* Correspondence: angelica.mazzolari@unimi.it

TABLE OF CONTENTS

Table S1. Top 25 features for the LOO validated model based on the MT-dataset and their feature importance values.

Table S2. List of 20 physico-chemical and stereo-electronic descriptors used to build the random forest models and for the PCA study.

Table S3. List of 127 BlueDesc descriptors used for the PCA study.

Table S4. Grid for hyperparameter optimization applied during the nested 5-fold cross-validation.

Descriptor	Feature importance
Lipole	0,011275776
WNSA-3	0,011372096
PNSA-3	0,011564167
Vdiam	0,01174551
ECCEN	0,012601431
XLogP	0,012943108
WTPT-4	0,013156247
ECFP343	0,013281842
joelib2.feature.types.LogP	0,014426853
joelib2.feature.types.PolarSurfaceArea	0,014908135
DIPOLE	0,015296358
IONIZATION_POTENTIAL	0,015894437
RPCS	0,015897675
HOMO_ENERGY	0,016552016
ECFP716	0,016991979
WTPT-3	0,01771452
ECFP150	0,017962269
RNCS	0,018679488
ATSc1	0,019366336
ATSc3	0,0205993
PNSA-1	0,022629303
Zagreb	0,027769799
WTPT-1	0,032494886
WNSA-1	0,036299698
CORE-CORE_REPULSION	0,0524351

Table S1: top 25 features for the LOO validated model based on the MT-dataset and their feature importance values.

**Name**	**Type of descriptor**	**Software**
PSA	Physico-chemical	VEGA ZZ
ASA		
SAS		
SAV		
Sdiam		
Vdiam		
Volume		
Gyrrad		
VlogP		
Lipole		
Molecular weight	Stereo-electronic	MOPAC 2016
Cosmo area		
Cosmo volume		
Total energy		
Electronic energy		
Homo energy		
Lumo energy		
Core-core repulsion		
Ionization potential		
Dipole		

Table S2: list of 20 physico-chemical and stereo-electronic descriptors used to build the random forest models and for the PCA study.

Name	Type of descriptor
joelib2.feature.types.TopologicalDiameter	Topological
joelib2.feature.types.GraphShapeCoefficient	
joelib2.feature.types.MolecularWeight	Constitutional
joelib2.feature.types.KierShape1	Topological
joelib2.feature.types.KierShape2	
joelib2.feature.types.KierShape3	
joelib2.feature.types.LogP	Constitutional
joelib2.feature.types.PolarSurfaceArea	Topological
joelib2.feature.types.GlobalTopologicalChargeIndex	
joelib2.feature.types.GeometricalDiameter	
joelib2.feature.types.GeometricalRadius	
WTPT-1	Topological
WTPT-2	
WTPT-3	
WTPT-4	
WTPT-5	
chi0vC	Topological
chi1vC	
chi0C	Topological
chi1C	
Zagreb	Topological
SCH-5	Topological
SCH-6	
VCH-5	
VCH-6	
SC-3	Topological
SC-5	
VC-3	
VC-5	
SP-0	Topological
SP-1	
SP-2	
SP-3	
SP-4	
SP-5	
SP-6	
SP-7	
VP-8	
VP-9	
VP-10	
VP-11	
VP-12	
VP-13	
VP-14	

VP-15	
SPC-4	Topological
SPC-5	
SPC-6	
VPC-4	
VPC-5	
VPC-6	
WPATH	Topological
WPOL	
ECCEN	Topological
ATSc1	Topological
ATSc2	
ATSc3	
ATSc4	
ATSc5	
ATSm1	Topological
ATSm2	
ATSm3	
ATSm4	
ATSm5	
bpol	Electronic
apol	Electronic
GRAV-1	geometrical
GRAV-2	
GRAV-3	
GRAVH-1	
GRAVH-2	
GRAVH-3	
GRAV-4	
GRAV-5	
GRAV-6	
LOBMAX	geometrical
LOBMIN	
MOMI-X	geometrical
MOMI-Y	
MOMI-Z	
MOMI-XY	
MOMI-XZ	
MOMI-YZ	
MOMI-R	
Wlambda1.unity	Topological
Wlambda2.unity	
Wlambda3.unity	
Wnu1.unity	
Wnu2.unity	

Weta1.unity	
Weta2.unity	
Weta3.unity	
WT.unity	
WA.unity	
WV.unity	
WK.unity	
WD.unity	
PPSA-1	Electronic and geometrical
PPSA-2	
PPSA-3	
PNSA-1	
PNSA-2	
PNSA-3	
DPSA-1	
DPSA-2	
DPSA-3	
FPSA-1	
FPSA-2	
FPSA-3	
FNSA-1	
FNSA-2	
FNSA-3	
WPSA-1	
WPSA-2	
WPSA-3	
WNSA-1	
WNSA-2	
WNSA-3	
RPCG	
RNCG	
RPCS	
RNCS	
THSA	
TPSA	
RHSA	
RPSA	
XLogP	Constitutional

Table S3: list of 127 BlueDesc descriptors used for the PCA study.

Parameter	Explored values for MQ-dataset	Explored values for MT-dataset
Max_features	50/100/200	20/70/120
N_estimators	20/70/120	20/50/100
Class_weight	0.5 : 0.5 / 0.3 : 0.7 / 0.1: 0.9	0.5 : 0.5

Table S4: grid for hyperparameter optimization applied during the nested 5-fold cross-validation.
